# Imaging the intracellular degradation of biodegradable polymer nanoparticles

**DOI:** 10.3762/bjnano.5.201

**Published:** 2014-10-29

**Authors:** Anne-Kathrin Barthel, Martin Dass, Melanie Dröge, Jens-Michael Cramer, Daniela Baumann, Markus Urban, Katharina Landfester, Volker Mailänder, Ingo Lieberwirth

**Affiliations:** 1 Max-Planck-Institute for Polymer Research, Ackermannweg 10, 55123 Mainz, Germany; 2 3rd Department of Medicine (Hematology, Oncology, and Pneumology), University Medical Center of the Johannes Gutenberg-University Mainz, Langenbeckstraße 1, 55131 Mainz, Germany

**Keywords:** biodegradation, mesenchymal stem cells, PLLA nanoparticles, transmission electron microscopy

## Abstract

In recent years, the development of smart drug delivery systems based on biodegradable polymeric nanoparticles has become of great interest. Drug-loaded nanoparticles can be introduced into the cell interior via endocytotic processes followed by the slow release of the drug due to degradation of the nanoparticle. In this work, poly(L-lactic acid) (PLLA) was chosen as the biodegradable polymer. Although common degradation of PLLA has been studied in various biological environments, intracellular degradation processes have been examined only to a very limited extent. PLLA nanoparticles with an average diameter of approximately 120 nm were decorated with magnetite nanocrystals and introduced into mesenchymal stem cells (MSCs). The release of the magnetite particles from the surface of the PLLA nanoparticles during the intracellular residence was monitored by transmission electron microscopy (TEM) over a period of 14 days. It was demonstrated by the release of the magnetite nanocrystals from the PLLA surface that the PLLA nanoparticles do in fact undergo degradation within the cell. Furthermore, even after 14 days of residence, the PLLA nanoparticles were found in the MSCs. Additionally, the ultrastructural TEM examinations yield insight into the long term intercellular fate of these nanoparticles. From the statistical analysis of ultrastructural details (e.g., number of detached magnetite crystals, and the number of nanoparticles in one endosome), we demonstrate the importance of TEM studies for such applications in addition to fluorescence studies (flow cytometry and confocal laser scanning microscopy).

## Introduction

Nowadays biocompatible and biodegradable polymers are customary materials in daily medical routine. By tailoring their macromolecular architecture, it is possible to precisely adjust mechanical properties as well as features for interaction with living organisms, for example, the decomposition dynamics of resorbable threads in surgery or the surface compatibility of bone implants. In particular, materials based on poly(ε-caprolacton) and poly(lactic acid) have found their way as resorbable materials into medical applications. Preferentially, the L-isomer of lactic acid is used, based on the fact that this form is naturally occurring and can be metabolized with no serious inflammatory effect on the surrounding tissues [1]. The decomposition of poly(L-lactic acid) (PLLA) takes place via hydrolysis when exposed to an aqueous environment and can be enzymatically catalyzed [2]. The hydrolysis of the ester groups is an autocatalytic process by carboxylic acid end groups [3–4]. The decomposition of macroscopic PLLA implants has already been shown in vitro and in vivo [5–8] and can be accelerated by the presence of enzymes or bacteria. It is speculated that L-lactic acid, which is the degradation product of PLLA, is transformed into water and CO_2_ via the citric acid cycle [9].

In addition to many other factors such as temperature or pH [10], the degree of crystallinity and the molecular weight of the PLLA affect the rate of hydrolysis [2,8]. To get a feeling for the degradation constant, Pistner et al. demonstrated that millimeter-sized samples of amorphous PLLA degraded in approximately one year in in vivo experiments [7].

Grizzi et al. studied the influence of sample shape and size on the non-enzymatic degradation comparing plates, millimetric beads, microspheres and cast films of PLLA [11]. By determination of the relative weight loss, they found that the bulkier samples degrade more rapidly (because the degradation rate is dominated by bulk disintegration processes) and surface hydrolysis seems to be considerably slower. A millimeter-sized plate was degraded by 50% of its initial weight within approximately 10 weeks in pH 7.4 phosphate buffer degradation medium, whereas a film of sub-millimeter thickness lost only 5% of its initial weight. This counterintuitive observation is explained by the entrapment of degradation products in the interior of the sample, which increases the local concentration of carboxylic end groups. When the surface-to-volume ratio is increased, surface-dominated processes increasingly contribute. Accordingly, particles with a size in the submicron domain can no longer be considered to undergo bulk degradation. This is because degradation products originating within the nanoparticle interior will no longer be entrapped, but rather are likely to diffuse out of the particle due to the short diffusion path. Stated simply, this means that significant bulk degradation of PLLA nanoparticles is not to be expected. Therefore, the degradation behavior of nano-sized PLLA materials should be very interesting as many drug delivery systems are currently being investigated for their use in vitro and in vivo*.*

Investigations of in vivo degradation of PLLA are currently restricted to at least micron-sized samples. These were applied to animal models and cell and bacteria cultures [1,12–13]. The number of studies demonstrating the intracellular degradation of polymeric material are, in contrast, outnumbered and accordingly few and far between. Akashi et al. incubated poly(γ-glutamic acid)-encapsulated ovalbumin nanoparticles into macrophage cells [14]. They used DQ ovalbumin, which is a self-quenched ovalbumin conjugate that exhibits fluorescence after proteolytic degradation. Following the degradation process, they employed confocal fluorescence microscopy and found that the degradation rate of smaller particles (40 nm) is lower than for larger ones (200 nm). However, the degradation was observed only 180 min after incubation into the cells.

In general, the quantification of nanoparticle uptake and cell loading can be classified into sample-conserving and sample-destructive techniques. The latter group typically comprises techniques such as mass spectroscopy, field-flow fractionation or radioactive labelling, whereas conserving techniques are usually based on microscopic methods. A good comparison of these different quantification methods is given by Elsaesser et al. [15].

For studying the intracellular degradation process, the polymer nanoparticles must be introduced into the cell and observed over a long period of time. PLLA nanoparticles are an ideal candidate for this purpose due to their potential to be taken up by the cell via endocytotic processes [16]. Furthermore, biodegradable polymeric nanoparticles are a promising vehicle for smart drug delivery systems and to this regard, it is even more important to examine intracellular degradation dynamics of these bio-polymers.

The objective of this work is to follow the fate of intracellular PLLA nanoparticles over a long time period of 14 days, primarily by means of transmission electron microscopy (TEM), in order to demonstrate their degradation. Furthermore, confocal laser scanning microscopy (CLSM) and flow cytometry were used to monitor the nanoparticle load of individual cells. As a probe we chose tailor-made PLLA nanoparticles marked with a fluorescent dye for fluorescence-based measurements, and additionally decorated with magnetite nanoparticles on the surface [17]. Hydrolysis of the PLLA releases the magnetite nanoparticles, serving as an indicator for PLLA degradation. As a model cellular system, mesenchymal stem cells (MSCs) were chosen because they are promising candidates for regenerative medicine [18–19] and they show a moderate cleavage rate without addition of transfection agents or mitotic inhibitors [20–21].

Common strategies to monitor and quantify the nanoparticle load on a single cell level are based on detecting fluorescently labeled nanoparticles on a single cell level using time- and space-resolved microscopy-based techniques [22–23]. However, these techniques simply quantify the average number of nanoparticles per cell. For our purpose, however, the local release of magnetite from the PLLA nanoparticles is of relevant importance rather than the total number of PLLA particles per individual cell. Similar to the analysis of uptake experiments from Brandenberger et al. [24], our investigations were dependent on the analysis of highly spatially resolved TEM micrographs and their subsequent statistical analysis. In contrast, however, we use perfectly tailored nanoparticles that will release magnetite upon degradation of the PLLA, which is evaluated by means of statistical interpretation of TEM micrographs.

## Experimental

### Preparation of nanoparticles

The nanoparticles used in this study were composed of poly(L-lactic acid) (PLLA), decorated with approximately 25 nm-sized magnetite nanoparticles, prepared by combining miniemulsion and emulsion–solvent evaporation techniques. The particles were labeled with the fluorescent dye, *N*-(2,6-diisopropylphenyl)perylene-3,4-dicarboximide (PMI). A detailed description of the preparation can be found elsewhere [17].

### Human mesenchymal stem cell cultivation

Human MSCs were generated from bone marrow aspirations or explanted hips after obtaining informed consent in accordance with the terms of the ethics committee of the University of Ulm, Germany. Primary human MSCs were generated as previously described [25] and kept in alpha minimum essential medium (α-MEM, Lonza, Belgium) supplemented with 20% fetal calf serum (FCS), 100 units of penicillin, 100 μg·mL^−1^ streptomycin, and 1 mM pyruvate (all from Invitrogen, Germany) in addition to 3 mL of ciprofloxacin (Fluka, Switzerland; 2 mg·mL^−1^, 0.6%). Cells were grown in 500 cm^2^ triple flasks (Nunc, Germany) in a humidified incubator at 37 °C and 5% CO_2_. The culture medium was changed twice a week. At confluence, cells were detached by 0.5% trypsin (Invitrogen, Germany) and seeded in the specified concentrations.

### Flow cytometry

Flow cytometry was used for quantification of intracellular nanoparticles and for the analysis of cell viability. Similar to the procedures previously described [26], adherent cells were detached by trypsin (Gibco, Germany) and seeded in α-MEM at a density of 100 000 cells per well in 6-well plates (Greiner, Germany). On the following day, the PLLA particles (labeled with 0.428 mg·g^−1^ polymer PMI) were added at a concentration of 300 μg·mL^−1^ to the medium. After an incubation time of 24 h, the supernatant was removed, washed with PBS (phosphate buffered saline) and fresh medium was added to the cells. At specified times (directly after incubation at 24 h, 48 h, 72 h, 5 days, 7 days, and 14 days), the cells were prepared for flow cytometry experiments. Cells were confluent and split at days 4 and 11, and newly seeded at a density of 100 000 cells/well in a 6-well plate.

To prepare the cells for analysis, the following steps were performed. First the cells were washed with PBS, then trypsinized, centrifuged (3 min, 1500 rpm) and stained with 0.2 mg·mL^−1^ 7-aminoactinomycin D (7-AAD, Sigma-Aldrich) as a fluorescence apoptosis marker for 15 min at room temperature in the dark before the cell pellet was finally re-suspended in PBS. Flow cytometry measurements were performed with a CyFlow ML using FlowMax 2.57 software (Partec, Germany). The FL1 channel (488 nm laser) was used to analyze the uptake of nanoparticles and FL6 (561 nm laser) was used for 7-AAD measurements. For the analysis, the cells were selected on a forward scatter/sideward scatter plot, thereby excluding cell debris. These gated events were then further analyzed for the FL1 and FL6 channels. The median intensity in the FL1 was determined from 2D histograms. This corresponds to the number of nanoparticles taken up by or associated with individual cells. For 7-AAD, the events in the cell gate were analyzed on a FL1/FL6 dot-plot for FL6 fluorescence and three different populations (viable, apoptotic, dead) were determined by using negative controls and the apoptotic and dead cells present in cell cultures. All values are triplicates with the error bars representing the standard deviation.

### Confocal laser scanning microscopy (CLSM)

Confocal laser scanning microscopy (CLSM) was applied to demonstrate the intracellular distribution of nanoparticles over the period of 14 days. As described in [26], for confocal laser scanning microscopy (CLSM), MSCs were seeded in α-MEM solution at a density of 20 000 cells in ibiTreat µ-slides (IBIDI, Germany). On the following day particles were added to the medium at a concentration of 300 μg·mL^−1^. After an incubation time of 24 h, the supernatant was removed and fresh medium was added. Cells were confluent and split at days 4 and 11, and newly seeded out in a density of 20 000 cells/ibiTreat µ-slide.

At the specified measurement times the medium was again removed. Before analysis, the cells were washed two times with PBS. The images for the intracellular localization of the particles were taken using a commercial setup (LSM SP5 STED Leica laser scanning confocal microscope, Leica, Olympus, Germany), consisting of an inverse fluorescence microscope (DMI 6000 CS) equipped with a multi-laser combination, in addition to five detectors operating in the range of 400–800 nm. An HCX PL APO CS 63×/1.4–0.6 oil-immersion objective was used in these studies. Fluorescent particles were excited with an Ar laser (≈20 mW, λ = 488 nm), and detected at 510–550 nm, which corresponds to green in color. The membrane of the MSCs was stained with CellMask™ Orange (2.5 μg·mL^−1^, Invitrogen, Germany), which is pseudocolored in the images as red surrounding the cytoplasm (excited with a DPSS 561 nm laser (≈20 mW) and detected at 580–620 nm). The nucleus was stained with DraQ5 (2.5 μM, Biostatus, U.K.) and appears as a blue color (excited with a HeNe laser at 633 nm, ≈10 mW and detected at 680–750 nm).

### Transmission electron microscopy (TEM)

TEM was performed to precisely localize the intracellular particles and characterize morphological changes. For preparation, cells were seeded out in a 24-well plate (Greiner, Germany) containing three sapphire disks, surface C-coated. Cells were seeded out and adhered for 24 h on the sapphire discs. PLLA particles were added at a concentration of 300 µg·mL^−1^ to the cell medium and incubated for 24 h. After incubation, the supernatant was removed, the cells were washed with PBS and new medium was added. At days 4 and 11 after cell seeding, the cells were detached by trypsin and newly seeded out.

At the specified residence times, the sapphire disks were removed from the wells and dipped into 1-hexadecane to remove the remaining medium. The disks were covered with aluminum disks to prevent squeezing of the cells and the sandwich was injected into a high pressure freezing device (Wohlwend, Switzerland). The frozen sandwich was stored in liquid N_2_.

The aluminum cover was removed in a liquid N_2_ environment and the sapphire disk was transferred into anhydrous acetone at −90 °C, (Merck, Germany) containing 4% aqueous osmium tetroxide (Roth, Germany) and 0.1% uranyl acetate (Merck, Germany) as a freeze substitution (Leica EM AFS2, Germany).

The samples were slowly warmed to 0 °C over a time period of 18–20 h. After 1 h the freeze-substitution samples were warmed to room temperature, then the substitution medium was removed and the disks were washed two times with an aliquot of acetone. The disks were incubated in 1:1 EPON^®^ for 3 min an acetone solution and left over night in a 100% EPON^®^ solution (epoxide resin, Fluka, Switzerland). The next day, infiltration was completed and the disks were transferred into a new 1.5 mL reaction tube containing fresh 100% EPON^®^ and arranged with the cell covered site to the vessel opening. The tubes were left in a furnace at a temperature of 60 °C for 3 days to polymerize the epoxide resin. The hardened samples were quickly cooled down with liquid N_2_, in order to break the resin block at the interface to the disk. The cells were then enclosed in the resin block. The block was divided in halves, then trimmed into a trapezoid area with an abundant number of cells, and then fixed in the ultra-microtome (Leica Ultracut UCT). With a diamond knife (Diatome Ultra, Switzerland), 60 nm thick sections were achieved and applied on a copper grid (3.05, 300 mesh, Agar Scientific, U.K.).

The sections were observed in a Zeiss EM 912 transmission electron microscope with a tungsten filament at an acceleration voltage of 120 kV. Micrographs were taken via a Cantega Olympus slow scan CCD camera.

### UV−visible (UV−vis) spectroscopy

A Lambda 25 (Perkin-Elmer) UV−vis spectrometer was used to monitor the formation of the FeCl(H_2_O)_5_^2+^ complex upon addition of HCl to magnetite particles. The FeCl(H_2_O)_5_^2+^ complex shows a characteristic absorption band at 340 nm. The cell was filled with 3 mL of diluted HCl (28 wt %) for background measurements. Subsequently, 75 µL of sample solution (10% solid content) was added to the cell and the adsorption at 340 nm was monitored for approximately 30 min. The purpose of this measurement was to compare the accessibility of iron for the HCl in pure magnetite solution and in the PLLA-magnetite composite particles.

## Results and Discussion

Following the uptake of polymer nanoparticles into cellular compartments and their subsequent residence, observation at various length scales is required, yielding different information. Moreover, for biodegradable nanoparticles, the verification of their decomposition within the cell by means of image-based examination such as transmission electron microscopy might be problematic. In order to cope with these problems we used tailor-made, PLLA nanoparticles containing a fluorescent marker (PMI) suitable for flow cytometry and laser scanning microscopy (LSM) measurements. Additionally, these particles were decorated with magnetite nanocrystals that were encapsulated inside the polymer. The detection of free magnetite nanocrystals within the cell will accordingly serve as indicator for the decomposition of the PLLA nanoparticle. In the following, we will consider a magnetite nanocrystal to be “free” if it is not obviously attached to a PLLA nanoparticle, that is, if the separation is more than twice the nanocrystal diameter.

In our experimental outline the MSCs were incubated with the fluorescent-magnetite-labeled PLLA nanoparticles for a total time of 24 h during which uptake took place. After this incubation time, the incubation medium (including the nanoparticles) was removed and replaced by a medium with FCS but without nanoparticles. Subsequently, the first samples were prepared for FACS, LSM and TEM measurements. Further sampling times thereafter were set to 48 h, 72 h, 5 days, 7 days, and 14 days after the start of incubation. This rather long incubation time was a compromise with the TEM examination. The probability of finding nanoparticles within the cell by means of TEM is quite low since the preparation yields an approximately 60 nm-thick cross section through the cell. This cross section must coincide with the desired nanoparticles in order to be observed. Additionally, every mitosis of the cell will further decrease the observation probability of incorporated nanoparticles during TEM examination. Hence, the initial density of incorporated nanoparticles was chosen to be as high as possible. Accordingly, the amount of PLLA particles that are taken up by the cells must be maximized with a long incubation time.

Because the TEM measurements cannot reflect the precise number of polymer nanoparticles contained within the cells, additional flow cytometry and confocal laser scanning microscopy investigations were conducted. The flow cytometry yields information about the median fluorescence intensity of the MSCs. Hence, it allows for tracking of the average quantity of incorporated PLLA nanoparticles over time in the cells. Additional CLSM measurements complement the overall picture, detecting and imaging the PLLA nanoparticles within several individual cells, but without the possibility to determine their degree of degradation.

### Flow cytometry

**Figure 1:**
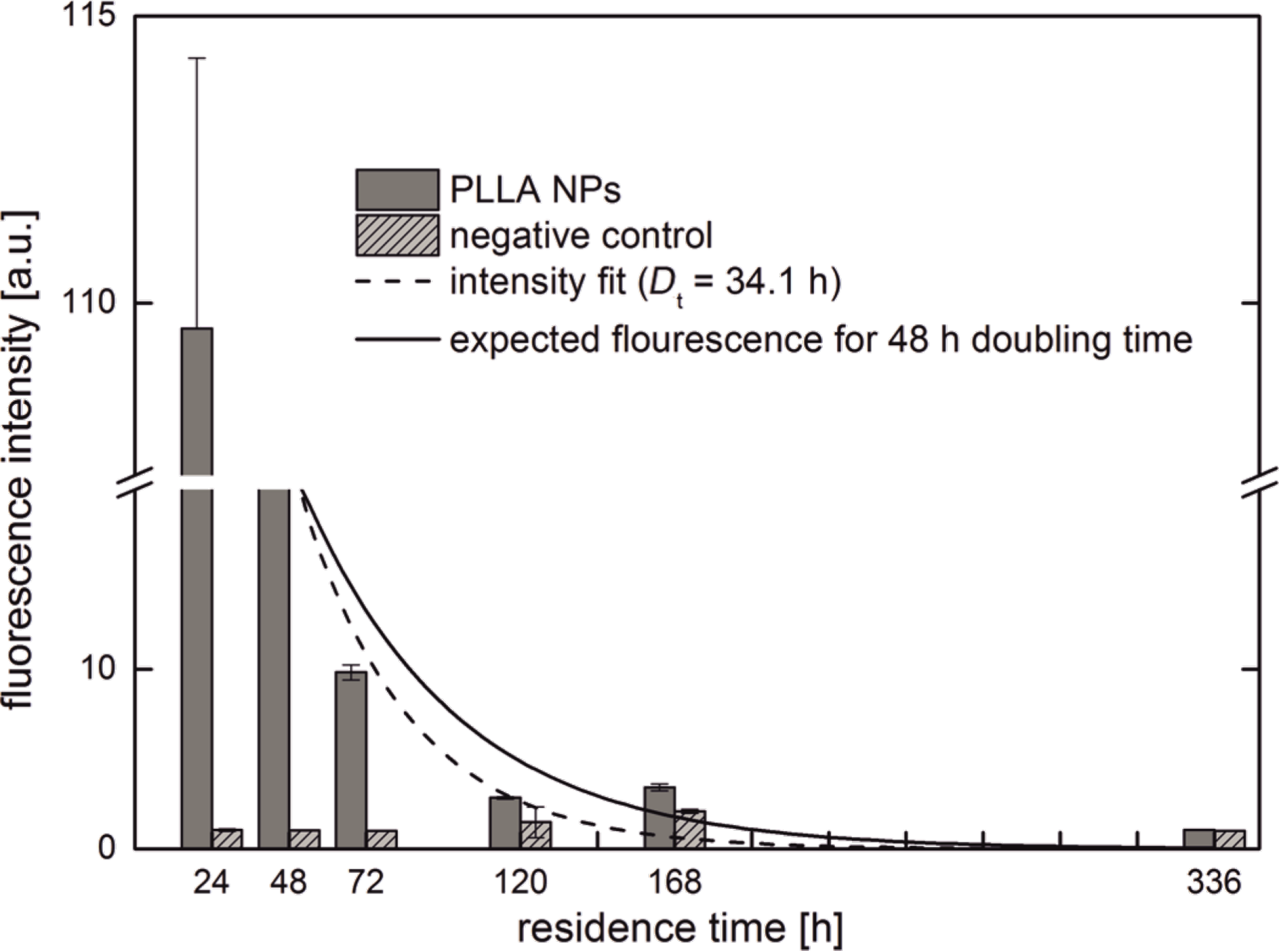
Flow cytometry measurements showing the relative fluorescence intensity of the cells for different residence times.

The number of polymer nanoparticles adhering to or taken up by the MSCs was analyzed quantitatively by flow cytometry by monitoring the intensity of the fluorescence signal of the fluorescently labeled (PMI as a dye) PLLA particles. Following the residence of the PLLA particles within the MSCs, these were sampled at several predefined periods after the start of the incubation process. The intensity of the fluorescence signal as a function of time is shown in Figure 1. As expected, the fluorescence intensity decreases with increasing time from the initial 24 h incubation. After 14 days the measured fluorescence intensity reached a level equal to the negative control and the PLLA nanoparticle concentration in the MSCs decreased below the detection limit. However, just after the end of the incubation time, the median fluorescence intensity of the MSCs is observed to be much higher than after another 24 h later (48 h after starting the incubation process). The measured fluorescence intensity shows the largest decrease after the end of incubation at 24 h. This has been observed in previous studies [27]. One explanation can be traced back to the fact that after removal of the incubation medium, excessive exocytosis of PLLA particles occurs [9]. Furthermore, Panyam et al. investigated the influence of bovine serum albumin (BSA), which is a main component of FCS that is added to the cell medium. The results showed that BSA significantly increased the exocytosis of the tested PLGA nanoparticles [28]. Given that we used FCS in the cell medium, the high exocytosis might be responsible for the significant decrease in the measured fluorescence intensity between 24 h and 48 h.

In addition to this initial sharp decrease in intensity (which is highly suspicious of exocytosis), there are two additional cellular processes that contribute to a decrease in concentration of PLLA nanoparticles within the cell: decomposition of the PLLA nanoparticles and mitosis. The latter process will reduce the average fluorescence intensity of the cell culture by a factor of two for every cell division. The individual PLLA nanoparticle distribution among the two daughter cells, however, might be highly asymmetric [29]. Hence, the average fluorescence intensity, and with that the mean PLLA particle concentration in the MSC culture, will follow an exponential decay law with a decay rate inversely proportional to the negative of the cell doubling time *D*_t_,

[1]
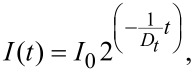


where *I*(*t*) and *I*_0_ are the average fluorescence intensity per cell at times *t* and *t = 0*, respectively. In the case of exocytosis of the fluorescently labeled PLLA particles (as observed between the end of the incubation time (24 h after starting the cell treatment) and the second sampling (48 h after starting the cell treatment)), this simple equation cannot be applied to fit the data. Assuming that this observed excessive exocytosis decreases during the subsequent 24 h, Equation 1 can be normalized to the fluorescence intensity measured 48 h after the start of the incubation time. We measured the doubling time of the MSCs with and without the presence of the PLLA nanoparticles to be 48 h and 42 h, respectively, which is in good accordance with the literature [30–31]. Using a cell doubling time, *D*_t_, of 48 h for MSCs gives the expected fluorescence intensity if only mitotic processes contribute to the decrease of intracellular nanoparticle concentration (solid line in Figure 1). On the other hand, one might fit Equation 1 to the fluorescence intensity data yielding a cell doubling time of *D*_t_ = 34.1 h (dotted line in Figure 1). Finally, 336 h after the start of incubation, the fluorescence intensity has decreased to a level equal to the negative control.

Additionally, flow cytometry measurements revealed that the same magnetite-labeled PLLA particles did not affect the viability of the MSCs. This has been shown in our previous works [27]. PLLA particles with an even higher iron concentration did not affect the cell viability over a period of 6 days.

### Confocal laser scanning microscopy (CLSM)

**Figure 2:**
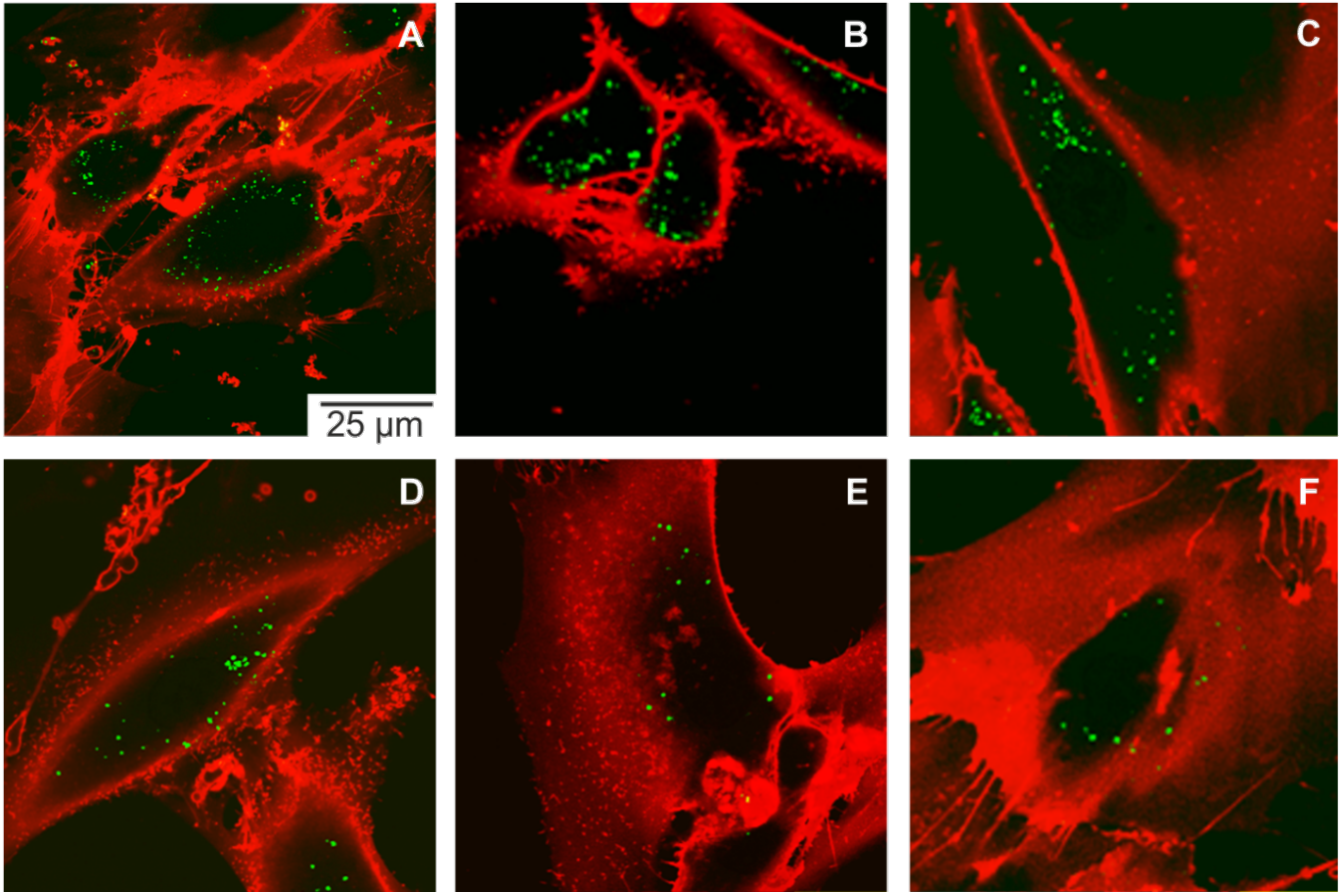
CLSM images of cells treated with PLLA/magnetite particles; incubation time: 24 h; pictures taken at specified times after particle addition: A) 24 h, B) 48 h, C) 72 h, D) 5 d, E) 7 d, and F) 14 days. Red: cell membrane; Green: PLLA nanoparticles.

In order to investigate the intracellular localization of the PLLA nanoparticles, CLSM investigation of the cell culture was conducted concurrently with the flow cytometry measurements. Figure 2 shows the CLSM micrographs of the MSCs at different times after starting the nanoparticle incubation. CellMask™ Orange was used to stain cell membranes and discriminates the extracellular from intracellular area. It is pseudocolored red, while particles with the fluorescent dye PMI are shown in green. DraQ5 ™ marks the nucleus and is pseudocolored blue. PLLA nanoparticles attached to the cell membrane are recognized by the overlay of red- and green-stained regions (cell membrane and PLLA nanoparticles, respectively) and are displayed as yellow pixels in the CLSM overlay images. However, there are no prominent attachments of the particles to the cellular membrane and only rarely are yellow pixels seen in the overlay image. Moreover, the observation of PLLA nanoparticles even after the longest residence time shows that the fluorescent dye is not released through diffusion but resides within the PLLA until its complete degradation. Compared to the negative control (untreated MSCs), the uptake of PLLA/magnetite NPs in the cells does not lead to any morphological changes of the cells over the whole observation period. Cell morphology is not affected by the incubation.

Generally, the particles were located inside the cells. After 24 h (Figure 2A), the PLLA nanoparticles were observed in a large amount in nearly every cell. Particle uptake appears to be evenly spread amongst all the cells. With increasing residence time, the intracellular particle content decreases (Figure 2B–F). However, with increasing time the particle distribution appears to be asymmetric. Some cells still show a relatively large number of incorporated nanoparticles, whereas in other MSCs nearly no external particles are present. Accordingly, this suggests that mitosis of MSCs leads to an asymmetric distribution of the incorporated nanoparticles to the two daughter cells.

### Transmission electron microscopy (TEM)

Transmission electron microscopic investigations per se reflect only a small section of the sample entity. To achieve a reasonably representative picture, a multitude of TEM micrographs should be taken into consideration. This becomes of special relevance when dynamic and/or biological issues come into focus in TEM examination. To examine the detailed morphology and the intracellular environment of the PLLA nanoparticles, however, a high resolution imaging technique such as TEM is indispensable.

#### Morphology of pristine PLLA nanoparticles

**Figure 3:**
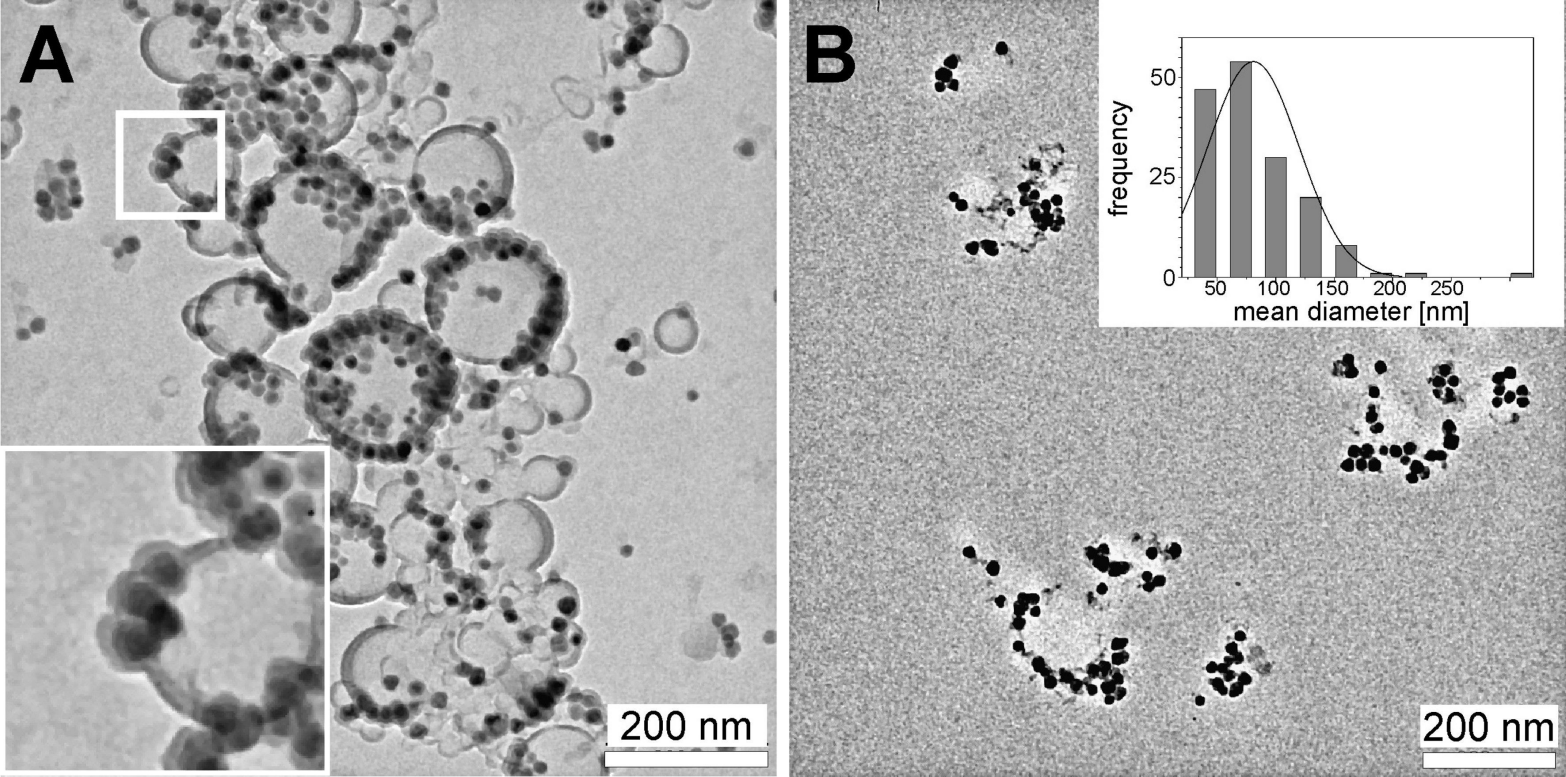
TEM bright field micrographs of a dispersion of magnetite-decorated PLLA nanoparticles prepared by drop casting and subsequent carbon evaporation (A) and by high pressure freezing (HPF) followed by freeze substitution and microtomy (B). The inset of B shows the distribution of PLLA nanoparticle diameters as determined from 164 individual particles after HPF processing and TEM examination.

To determine the initial morphology, size and the decoration quality, pristine magnetite-labeled PLLA nanoparticles were subjected to TEM investigation. Figure 3 shows TEM micrographs of the magnetite-decorated PLLA nanoparticles prepared by either drop casting on a carbon support film and additional carbon evaporation (Figure 3A), or prepared by high pressure freezing (HPF) followed by freeze substitution (Figure 3B). The latter preparation is identical to the preparation protocol of the MSCs, hence depicting the morphology of the PLLA nanoparticles as it is expected for intracellular observation, too. PLLA is an electron beam-sensitive polymer. It decomposes under irradiation into small, volatile hydrocarbon molecules until the majority of the material is decomposed. However, additional carbon evaporation can at least preserve the topography of the vanishing particles. This finally yields a TEM micrograph that looks like an image of capsular objects (Figure 3A) although the PLLA particles were initially compact spherical particles. Due to this material loss, areas containing PLLA appear bright in the TEM micrographs. The magnetite, on the other hand, appears as a dark contrast because of its high electron density (atomic number). The diameter of the PLLA particles imaged in Figure 3A ranges from 20 to 175 nm. This is in good agreement with the dynamic light scattering (DLS) measurement, which yielded an average diameter of 121 nm. The majority of the magnetite nanocrystals are attached to the PLLA particles. Only very few detached magnetite nanocrystals can be found.

**Figure 4:**
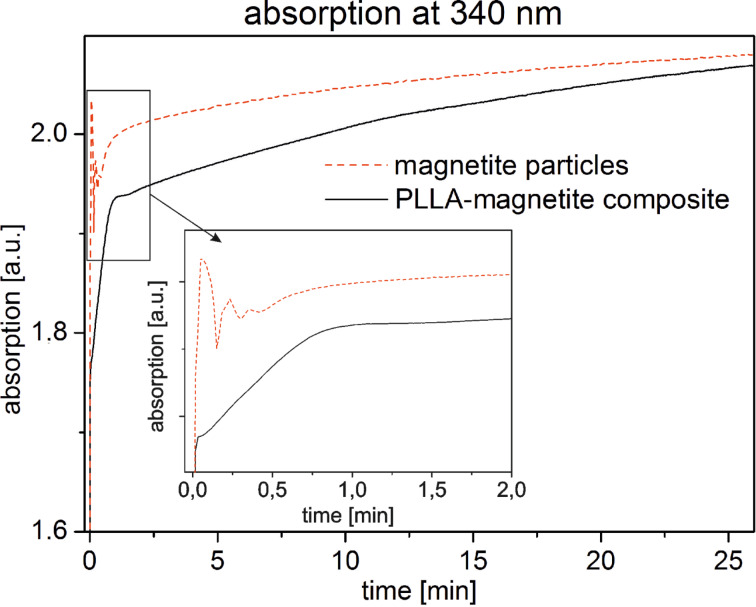
Time-dependent UV**–**vis adsorption measurement monitoring the formation of the FeCl(H_2_O)_5_^2+^ complex upon addition of HCl to pristine magnetite particles and magnetite-decorated PLLA particles. The formation process in case of the PLLA–magnetite particles is slightly retarded, indicating that an additional PLLA layer is covering the magnetite.

Furthermore, the attached magnetite nanocrystals exhibit an additional adlayer of approximately 5 nm thickness (inset of Figure 3A). Supposedly, this is a PLLA layer. In order to corroborate this morphological observation, we monitored the formation of FeCl(H_2_O)_5_^2+^ complex upon addition of HCl to pristine magnetite particles and magnetite-decorated PLLA particles by UV–vis adsorption spectroscopy. An additional PLLA layer should yield some protection and retard the complex formation, as documented in Figure 4. The effect is clearly visible, although it is not as strong as one would expect. But a 5 nm layer of PLLA cannot be considered as a diffusion barrier in this case and so the UV–vis result reasonably proves the existence of the additional layer covering the magnetite particles. Accordingly, the magnetite particles are intimately connected to the PLLA particle.

When prepared by HPF, freeze substitution and microtomy (which is the preparation protocol used to prepare the MSCs for TEM inspection throughout this work), the appearance of the pristine PLLA nanoparticles changes significantly (Figure 3B). Accordingly, Figure 3B reflects the morphological appearance of PLLA nanoparticles in an intracellular environment as imaged by TEM. The PLLA is not stained by OsO_4_ nor UrAc and due to the decomposition in the electron beam, PLLA-rich areas appear bright. One must keep in mind that here an approximately 60 nm thin cross section through the specimen is being imaged. Accordingly, the observed particle diameter of an individual object might not reflect its real dimensions, but more likely, a somewhat smaller value. This simply depends on the position of the object with regards to the “height” of the cross section – it may either image across the center of the particle or its cap only. Hence, the size distribution is shifted to smaller values (inset of Figure 3B). The maximum diameter of the observed PLLA nanoparticle was found to be about 80 nm.

The magnetite decoration of the pristine PLLA does not uniformly cover the surface, but rather tends to show some clustering. However, this does not exclude them as markers for our purposes.

#### Morphology and environment of intracellular PLLA nanoparticles

In order to identify PLLA nanoparticles within a thin section of a cell, we expect to observe similar features as pointed out above. The PLLA will appear bright and the magnetite as dark, approximately 20 nm-diameter particles. It is known that a cell can engulf nanoscale extracellular matter by endocytosis and formation of endosomes [32–35]. Hence, examination by TEM will not only focus on the localization of intracellular PLLA nanoparticles, but will contain information regarding the morphology of their surroundings as well.

**Figure 5:**
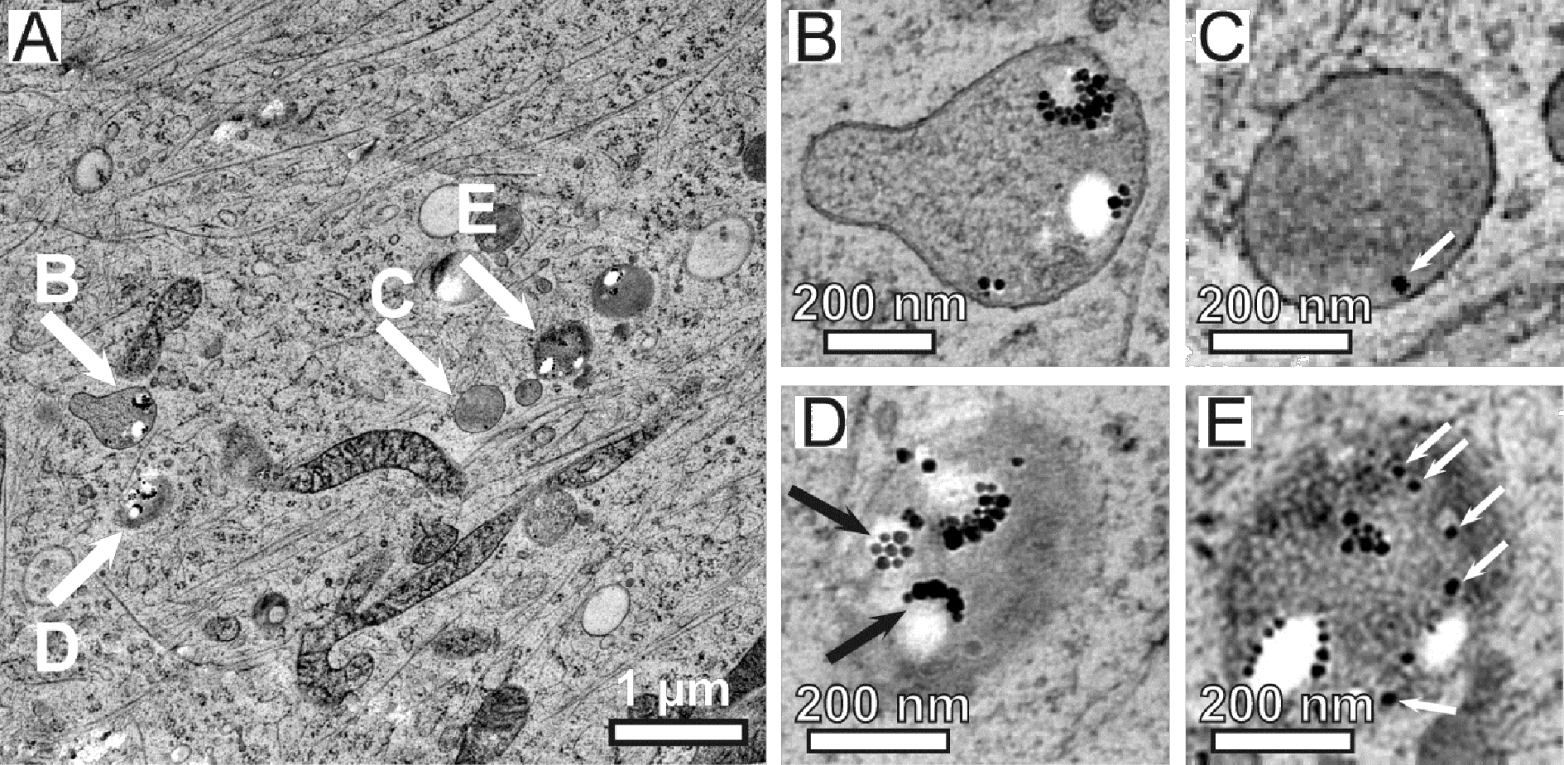
TEM bright field micrograph showing an overview (A) of a thin section of a MSC after 24 h of incubation with magnetite-decorated PLLA nanoparticles. Enlargement of endosomes, which contain extracellular material such as magnetite or PLLA are shown in (B–E).

Even 24 h after the start of incubation, the TEM observations of intracellular PLLA nanoparticles reveal a multitude of morphologies. Figure 5 gives an excerpt of some of these showing an overview (Figure 5A) of an area that contains several endosomes with extracellular material (marked by arrows and enlarged in Figure 5B–E). In some of the endosomes the outer membrane is clearly visible (Figure 5B and C), whereas sometimes it is not (Figure 5D and E). The latter can be attributed to a more or less tangential cut with respect to the membrane orientation. The appearance of the PLLA nanoparticles mainly depends on where the section circumscribes the nanoparticle. When only the cap of a PLLA nanoparticle is circumscribed by the section, one will find only a cluster of magnetite nanocrystals, whereas a more central cross section will reveal the true signature of the PLLA nanoparticle surrounded by some clusters of magnetite (Figure 5D, upper and lower black arrows). Accordingly, we will associate the observation of one or more magnetite cluster to the close vicinity of a PLLA nanoparticle, regardless if the PLLA itself is visible in the TEM micrograph. On the other hand, isolated magnetite particles as can be seen, for example, in Figure 5C and Figure 5E are marked with arrows that indicate intracellular PLLA decomposition. In the following we will define these isolated magnetite particles as “free magnetite” if no PLLA or magnetite particle can be found within a 20 nm radius.

**Figure 6:**
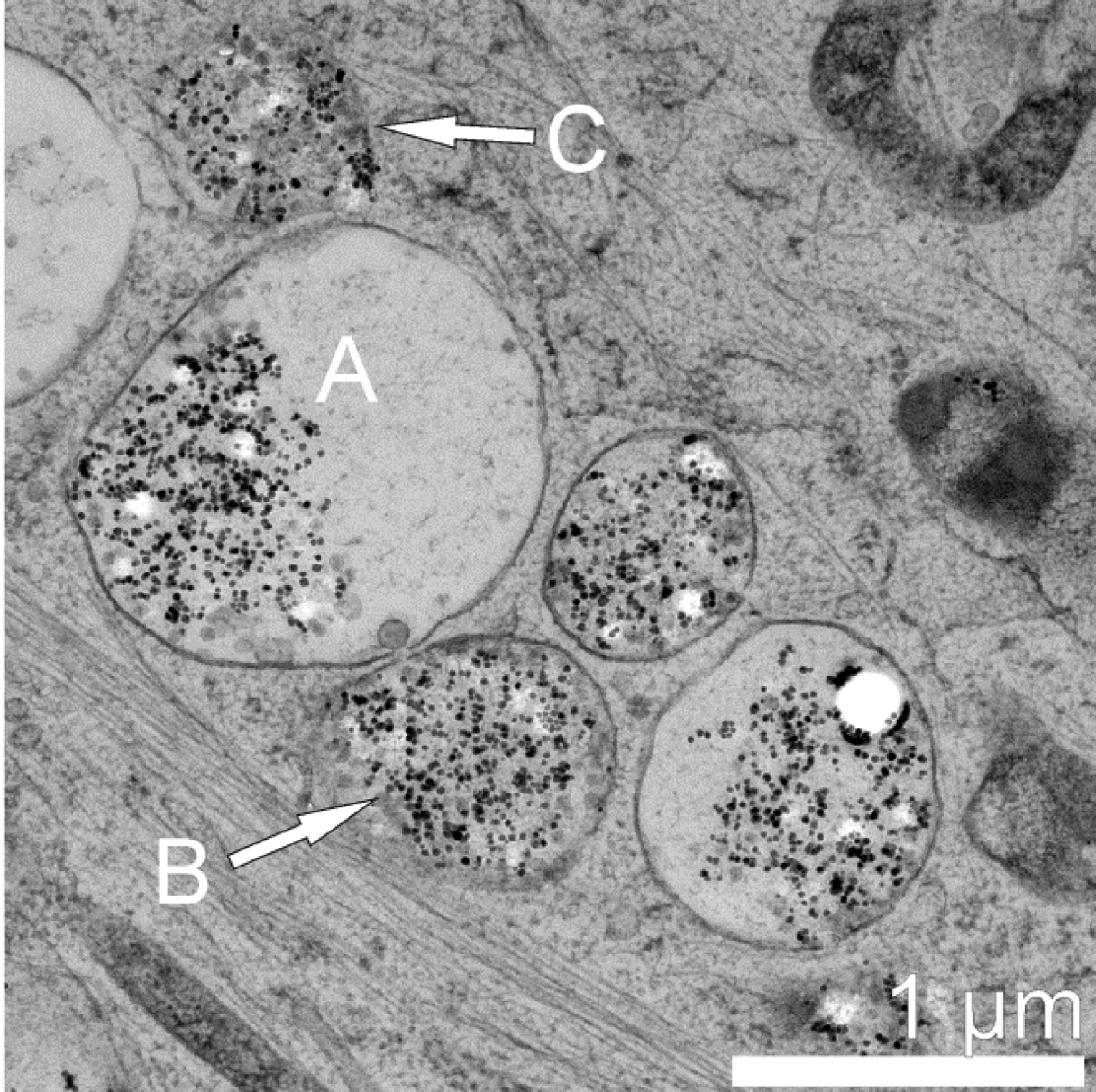
TEM bright field micrograph of a MSC after 24 h of incubation. The appearance of the PLLA containing endosome is quite different from those presented in Figure 4. Large endosomes filled with many magnetite and PLLA particles can be observed.

It is, to some extent, rather difficult to classify the entire variety of TEM observations. In total, we documented 132 endosomes containing PLLA nanoparticles and/or magnetite for the 24 h preparation. Figure 5 reflects only a small part of the observed endosome morphologies. Figure 6 displays a completely different appearance of PLLA containing intracellular endosomes. Large endosomes of 1 µm diameter and larger can be found. They show a bright contrast and are encircled by a membrane (Figure 6A). The external material, such as PLLA and magnetite, is concentrated to one side of this endosome. Many free magnetite particles can be found in this area. Additionally, there is one endosome completely filled with external material but with a discontinuous membrane (Figure 6, arrow B). Another endosome can be seen in the micrograph in Figure 6 (marked by arrow C) resembling the endosome marked B concerning the density and distribution of PLLA and magnetite particles, but without an observable surrounding membrane.

**Figure 7:**
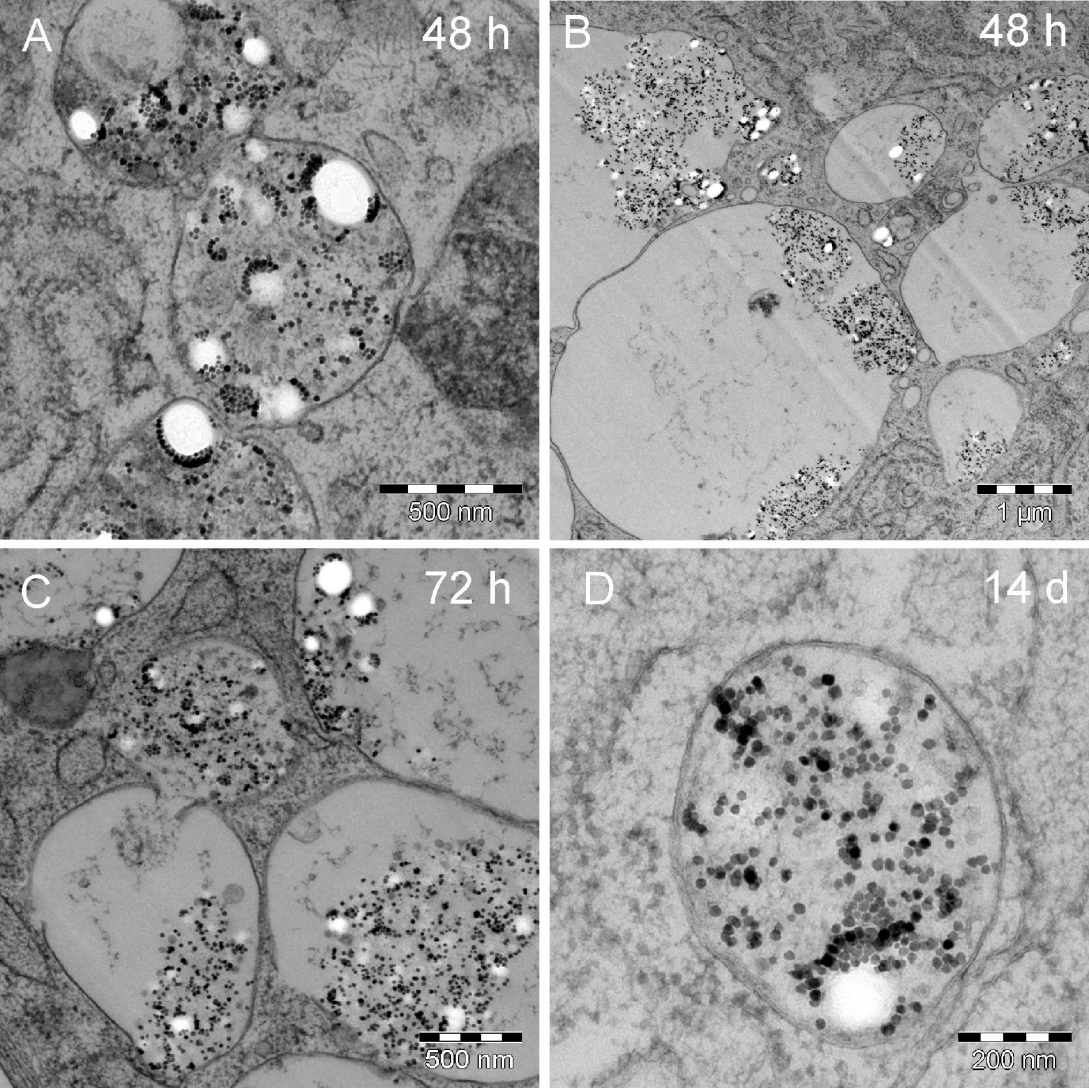
TEM bright field micrographs of endosomes observed in the MSCs at different residence times of PLLA nanoparticles. These images only provide an excerpt of the different endosomes observed for each observation time. At the 48 h time point, approximately 500 nm-sized endosomes become filled with external material (A). Additionally, “giant endosomes” were found (B). These could be found after 72 h as well (C) amongst others. Even after 14 days, the MSCs contain endosomes engulfing PLLA nanoparticles and magnetite nanocrystals (D).

48 h after the start of incubation (24 h incubation and another 24 h of PLLA nanoparticle residence in the MSC culture), the TEM examination again reveals a multiplicity of morphologically different PLLA–magnetite containing endosomes, as is the case for all residence times examined in this study. In total, we could identify 61 endosomes of interest for this time point. Figure 7A and Figure 7B show two exemplary micrographs which completely differ in the nature of the observed endosomes. On the one hand, smaller endosomes (approximately 500 nm in diameter) densely filled with external material (A) and on the other hand, “giant endosomes” exceeding several micrometers in diameter with irregularly distributed external material were observed.

This heterogeneous appearance continues throughout the entire observation time up to 14 days of residence. Giant endosomes coexist with smaller ones up to residence times of 7 days. Figure 7C displays this observation for a residence time of 72 h. After 14 days some of the inflated “giant endosomes” were still observable but the majority of external material was found in endosomes as shown for example in Figure 7D.

Morphologically, the PLLA nanoparticles as well as the magnetite nanocrystals were found grouped in cellular compartments, separated from the cytoplasm by a membrane or by the occurrence of a difference in contrast (e.g., Figure 6D). With increasing time after particle incubation, decreasing numbers of endosomes containing PLLA nanoparticles could be found. Over the course of the entire observation time (14 days), the PLLA nanoparticles and the magnetite nanocrystals could only be observed within endosomes and not within other cell compartments such as the cell nucleus, Golgi apparatus, or in mitochondria.

#### Quantitative analysis of TEM observations

**Figure 8:**
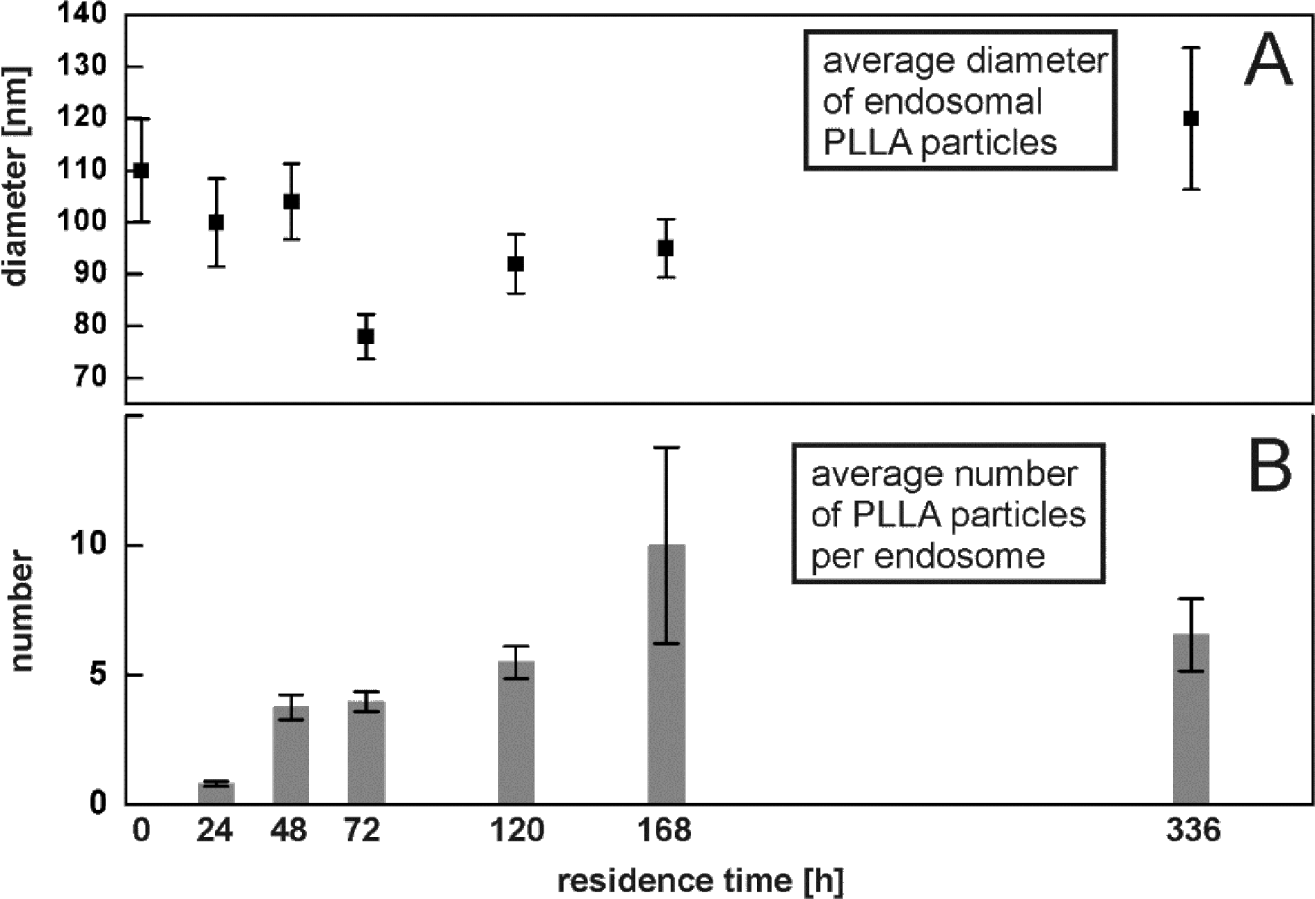
Quantitative evaluation of endosomal PLLA nanoparticles from TEM measurements. The average diameter of PLLA nanoparticles observed in HPF–freeze-substituted TEM samples (A). The first value at 0 h corresponds to the pristine PLLA nanoparticles. Number of PLLA nanoparticles averaged over the entity of all endosomes (B).

As stated above, TEM observations of external material inside MSCs are too heterogeneous to provide a coherent picture by presenting only an excerpt of micrographs. Accordingly, we comprised all endosomes of interest into a quantitative data collection. Each individual endosome was classified by six features: the number of visible PLLA particles, their mean diameter, the number of magnetite clusters, the number of free magnetite nanocrystals, the size of the endosome and the occurrence of a membrane. In this association an endosome becomes an endosome of interest when it contains any external material, for example, a PLLA nanoparticle, a free magnetite particle or a cluster thereof. Figure 8B shows the mean number of PLLA nanoparticles that was observed per endosome. However, one must keep in mind that only quasi-two-dimensional sections through the cell are observed. This means that the actual number of PLLA particles per endosome is likely much higher. However, one can observe a basic trend. The number of PLLA nanoparticles in the endosomes increases over time, which might indicate a fusion of endosomes. For example, such a fusion process is shown in Figure 7C, where one endosome appears to pour its content into a neighboring endosome. The average diameter of the PLLA nanoparticles is shown in Figure 8A. The first value at 0 h residence time corresponds to the measurement of the pristine PLLA nanoparticles as obtained from samples prepared by HPF and freeze substitution (see Figure 3B). The mean PLLA particle diameter decreases slightly with residence time, but 14 days after incubation, it shows a somewhat increased value compared to the pristine PLLA particles.

At this point it is appropriate to state some words on the statistics of this evaluation and the corresponding error bars. As valid for nearly all TEM measurements, the number of “events” (i.e., the number of PLLA nanoparticles or endosomes identified containing foreign material) is rather small. Especially for the longer residence time examinations the number of endosomes which were identified was low (for the 168 h and the 336 h, measurements only 7 and 22 endosomes were found, respectively). This is further complicated by the fact that in this case the TEM measurement is convolved with the broad PLLA particle size distribution (see inset in Figure 3) and the unknown cross section through the particle. Accordingly, Poisson statistics will take effect rather than a Gaussian standard deviation. The measurement error scales with *N*^−1/2^, where *N* is the number of events. Accordingly, this conservative and cautious error estimate leads to the large errors, especially for the long residence times.

**Figure 9:**
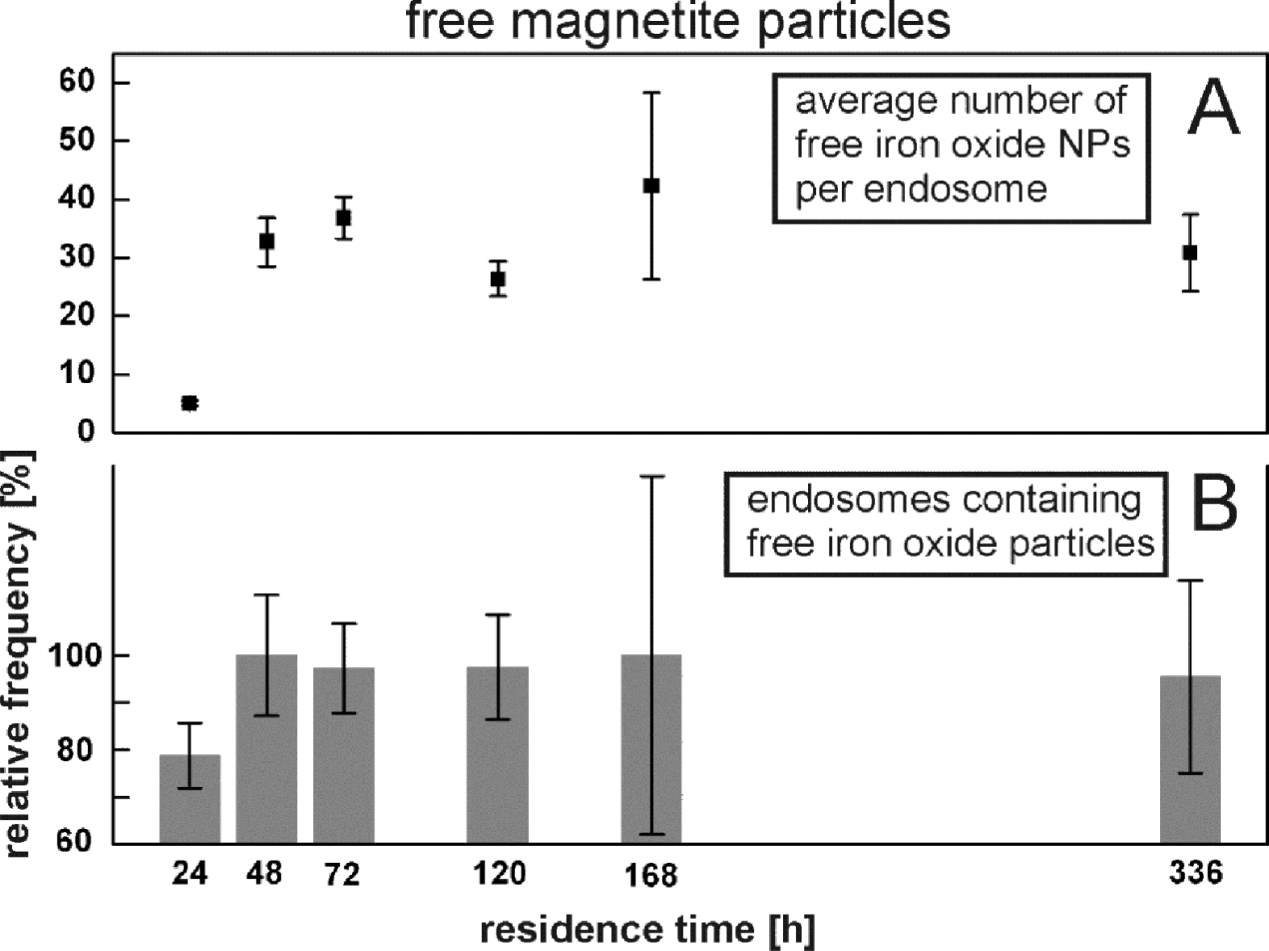
Quantitative analysis of TEM micrographs as to the occurrence of free magnetite nanocrystals. Average number of free magnetite nanocrystals per endosome (A) and relative proportion of endosomes containing at least one free magnetite nanocrystal (B).

The intracellular degradation of the PLLA nanoparticles was investigated by monitoring free magnetite nanocrystals within the cell. In this regard we consider a magnetite nanocrystal as free, if it is not visibly attached to PLLA (i.e., no PLLA or magnetite particle can be found within a 20 nm radius) and not clustered (e.g., free magnetite particles are marked by white arrows in Figure 5F). The average number of free magnetite particles per endosome increases significantly from 24 to 48 h of residence time, from 5 to 32 magnetite particles, respectively (Figure 9A), demonstrating that the release of these magnetite particles is rather fast and occurs within the first 24 to 48 h after uptake. Thereafter, no significant change is observed. The analysis of the TEM data regarding the fraction of endosomes that shows at least one free magnetite nanocrystal is shown in Figure 9B. At 24 h, approximately 80% of the endosomes contain at least one free nanoparticle. For all subsequent measurements, free magnetite was found in nearly every (100%) endosome of interest.

To summarize, our aim was to characterize the intracellular fate of PLLA nanoparticles that gain entry into MSCs with the focus on their degradation. For this purpose, we combined flow cytometry, confocal laser scanning microscopy, and transmission electron microscopy, where we aim to gain also quantitative data from these images. Understanding the dynamics of degradation and the intracellular trafficking might provide information as to whether these vehicles are suitable for intracellular drug delivery. Furthermore, detailed information about the cellular processes as seen from morphological examinations may give deeper insight into the cell strategies for handling the load of external NPs.

Proving the disappearance of something is challenging, especially for image-based techniques. One can do this, however, by using special markers such as self-quenched fluorescent dyes [14] or other non-degradable markers. We used magnetite nanocrystals for this purpose. Our tailor-made PLLA NPs were decorated with these magnetite particles and additionally fluorescently labeled with PMI for CLSM and flow cytometry measurements. The latter quantifies the average amount of fluorescence label per cell and hence yields a measure of the density of the material which is taken up. With time this density decreases due to at least three independent processes: exocytosis, mitosis and decomposition. The latter two should follow an exponential decay law with a characteristic decay constant. However, flow cytometry measurements are not suitable for discriminating the respective process responsible for the PLLA nanoparticle decrease directly. Assuming that mitosis is the dominating process after the initial exocytotic excess at 24 h, the exponential decay fit in Figure 1 (solid line) yields a somewhat larger signal than expected for the measured fluorescence intensity when compared to that measured. The majority of measured intensities lie below the expected values. Parallel to each cell experiment, the doubling time of our respective mesenchymal stem cells was determined to be 48 h in the presence of the respective PLLA nanoparticles. When fitting the flow cytometry data, the result is a decay constant of (34.1 h)^−1^ (see dashed line in Figure 1), which is much larger than expected from our preliminary cell doubling experiments. Accordingly, the MSCs lose NPs due to exocytosis or degradation processes. The biodegradation of PLLA is a quite slow process with a long half-life and retention as seen from intercorporal exposition experiments [5,7,36]. Here, retention times up to several months have been reported. On the other hand, exocytosis is a quite fast process (as compared to degradation) and it cannot be excluded that this is the origin for the observed decrease in fluorescence intensity. Accordingly, since the intracellular decay constant of PLLA nanoparticles should be very small, it might not be resolvable by flow cytometry time series experiments, because it may be superimposed by exocytosis processes with a much faster decay constant.

As already mentioned, in nano-sized particles, surface processes dominate due to the high surface-area-to-volume ratio. So most likely, degradation of any external, intracellular nano-sized object will propagate via its surface. Accordingly, with regards to the magnetite-decorated PLLA NPs incorporated into the MSCs, one should expect to observe a decrease in PLLA particle diameter accompanied by a release of free, unclustered magnetite nanocrystals in TEM examinations. Both are the case. The average diameter of PLLA NPs as determined from thin sections slightly decreases with residence time compared to the pristine particles (Figure 8A). However, for the largest residence time (336 h = 14 days), the mean PLLA diameter increased to a value way above the average pristine value. Any explanation for this is highly speculative. Swelling of the PLLA nanoparticles due to the absorption of water could be one reason. But one must keep in mind that the initial size distribution of PLLA NPs is rather broad and that selective removal of the small components will result in an increase of the average diameter, as observed in Figure 8A for 336 h. The large statistical error might be indicative of this. However, this interpretation contravenes with data from Grizzi [11] and Akashi [14] who found that larger PLLA particles tend to undergo faster extra- and intra-cellular degradation. On the other side, evidence of degradation of intracellular PLLA NPs is offered by the identification of free endosomal magnetite nanocrystals. Figure 9 shows two complementary statistical examinations of endosomal occurrence of detached magnetite nanocrystals from TEM micrographs. The average number of free magnetite per endosome significantly increases 48 h after the start of incubation, which is clear evidence of release from the PLLA NP surface within a short time frame (Figure 9A). Complementary, at the same time the number of endosomes that contain at least one free magnetite nanocrystal increases from 80% at 24 h after the start of incubation, to approximately 100% for all subsequent measurements (Figure 9B). In other words, initially 20% of the endosomes of interest do not contain any free magnetite nanocrystals. Consequently, this increase of free magnetite clearly rules out unbound magnetite from the incubation dispersion as the only source for the observation of free magnetite nanocrystals. For example, the endosome in Figure 5C may originate from the endocytosis of a single magnetite nanocrystal from the incubation dispersion. However, this is the only intracellular endosome we could find that contains exclusively one single magnetite particle.

Thus, endosomal PLLA NPs with magnetite decoration are the source of free magnetite particles within the endosomal cellular compartments. Keep in mind that the magnetite particles are initially covered with a thin layer of PLLA and thus intimately connected to the PLLA. Furthermore, the observation that the mean PLLA NP diameter decreases with increasing residence time leads to the conclusion that degradation of the PLLA must be the main source for the increase in the free magnetite concentration in the endosomes. Accordingly, we can “see” the intracellular PLLA NP degradation with increasing residence time.

Moreover, analyzing the vast array of TEM micrographs we observed a textbook-like evolution from smaller, early endosomes (Figure 5) (which contain only a few PLLA NPs) to a larger, late endosomes (which accumulate even more external material (Figure 6)) to the final lysosome. Additionally, we could also observe their fusion (Figure 7C). The development from the early endosomes to the much larger, late endosomes must take place within 24 h, as can be deduced from Figure 5 and Figure 6, which display different cells, but at the same point in time. Obviously, they differ in their development state, which can be attributed to the rather long incubation time of 24 h. This, in fact, generates a spectrum of development states such that one observes the coexistence of endosomes aged from (ideally) 0 to 24 h. Assuming that the larger endosomes in Figure 6 are the latter ones, one can deduce that degradation of the PLLA nanoparticles (observed by the increase of free magnetite in Figure 6) starts already within the first 24 h after endocytosis.

In correlation with the CLSM localization of the PLLA particles, the early endosomes are more or less homogeneously distributed over the intracellular cytoplasm (Figure 2A). A specific trafficking trend or a preferred location (e.g., near the nucleus) of the incorporated nanoparticles over the course of the experiment was not observed.

## Conclusion

We prepared tailor-made, PLLA nanoparticles decorated with magnetite nanocrystals and labeled with a fluorescence marker. Our results demonstrate that after uptake into MSCs, the PLLA nanoparticles undergo intracellular degradation. The incorporated nanoparticles enter the cell interior either individually or in small groups and terminate in cellular endosomes. By combining the results from flow cytometry, CLSM, and the statistics from a large number of TEM micrographs, we demonstrate the usefulness of this combined approach. The data from TEM analysis is especially helpful in gaining further insights into the ultrastructural processes. Even within 24 h of residence, a significant increase of released magnetite nanocrystals is observed, which is indicative of surface hydrolysis of the PLLA nanoparticles. However, the hydrolysis process of the whole PLLA nanoparticle is found to be rather slow because even after 14 days of intracellular residence, evidence of PLLA nanoparticles was still found within the cell.
